# Archaea predominate in the ammonia oxidation process in the sediments of the Yap and Mariana Trenches

**DOI:** 10.3389/fmicb.2023.1268790

**Published:** 2023-09-28

**Authors:** Hao Liu, Hongmei Jing, Fangzhou Wang

**Affiliations:** ^1^CAS Key Lab for Experimental Study Under Deep-Sea Extreme Conditions, Institute of Deep-Sea Science and Engineering, Chinese Academy of Sciences, Sanya, China; ^2^Southern Marine Science and Engineering Guangdong Laboratory (Zhuhai), Zhuhai, China; ^3^HKUST-CAS Sanya Joint Laboratory of Marine Science Research, Chinese Academy of Sciences, Sanya, China; ^4^University of Chinese Academy of Sciences, Beijing, China

**Keywords:** ammonia-oxidizing microbes, deep trench, sediments, potential ammonia oxidation rate, *amoA*

## Abstract

Ammonia-oxidizing archaea (AOA) and bacteria (AOB) play an important role in oxidizing ammonia to nitrite in different marine environments; however, their relative contribution to ammonia oxidation in the deep-sea sediments is still largely unknown. Sediment samples from seamounts and the Challenger Deep along the arc of the Yap Trench and the Mariana Trench were used for the investigation of the geographical distribution of AOA and AOB at the cDNA level, with associated potential nitrification rates (PNRs) being measured. AOA was predominated by *Candidatus Nitrosopumilus* and Nitrosopumilaceae, while *Methylophaga* was the major group of AOB. Significantly higher transcript abundance of the AOA *amoA* gene than that of AOB appeared in all samples, corresponding to the much higher RNRs contributed to AOA. Both the total and AOA PNRs were significantly higher in the deeper layers due to the high sensitivity of AOA to ammonia and oxygen than in AOB. In the surface layers, TN and TOC had significant positive and negative effects on the distribution of the AOA *amoA* gene transcripts, respectively, while ${\text{NH}}_{4}^{+}$ concentration was positively correlated with the AOB *amoA* gene transcripts. Our study demonstrated that AOA played a more important role than AOB in the ammonia-oxidizing process that occurred in the sediments of the Yap and Mariana Trenches and would expand the understanding of their ecological contribution to the nitrification process and nitrogen flux of trenches.

## Introduction

Nitrogen is the limiting nutrient in marine ecosystems, and ammonia oxidation, as the first and rate-limiting step of nitrification, is a very important process in nitrogen cycling in the ocean (Deutsch et al., 2007). This process was carried out by ammonia-oxidizing archaea (AOA) and ammonia-oxidizing bacteria (AOB) (Schleper and Nicol, 2010), which were frequently found in the marine sediments with different distribution patterns. For example, AOA dominated in the Ogasawara Trench (Nunoura et al., 2013), the Mariana Trench (Luo et al., 2015), the Eastern Indian Ocean (Wang et al., 2017), and Rushan Bay (He et al., 2018), while AOB dominated in the South Atlantic Ocean (Xu et al., 2014) and the Yangtze Estuary (Zheng et al., 2014). Variation in the predominance of the two groups seems impacted by salinity (Caffrey et al., 2007), ammonium concentration (de Gannes et al., 2014), dissolved oxygen concentration (Liu et al., 2011), and organic matter (Sun et al., 2013). In addition, the distribution of AOA and AOB was mainly studied on the surface sediments (Liu et al., 2018; Li et al., 2022); their vertical distribution within sediment was unclear. So far, their relatively ecological contribution to ammonia oxidation is still one of the most important issues related to the nitrogen cycle in the ocean deserving further investigation.

Potential nitrification rates (PNRs) could be applied to compare the different contributions of AOA and AOB with ammonia oxidation by estimation with the addition of excess ammonium under optimal conditions. Higher PNR contribution by AOB was found in the sediments of the mangrove (Li and Gu, 2013) and forest soil (Lu et al., 2015), while that by AOA had been reported from the sediments of the canal (Zhou et al., 2016), estuary (Li et al., 2015; Santos et al., 2018), and the deep ocean of the northeastern Japan Sea (Nakagawa et al., 2007). The heterogeneity might be explained by the concentrations of substrates and nutrients (Norman and Barrett, 2014), salinity (Santos et al., 2018), and soil pH (Enwall et al., 2005). In addition, previous studies on ammonia-oxidizing microorganisms in the oceanic sediments were mostly based on the DNA level (Xu et al., 2014; Lagostina et al., 2015; Luo et al., 2015; Wang et al., 2017), which reflected the total rather than those metabolic active assemblages, leading to sometimes contradictory findings. Quantifying the ammonia-oxidizing microorganisms at the RNA level with a combination of PNR measurements could effectively associate the active microbial proportions with their ecological functions.

Deep-sea environments are characterized by high hydrostatic pressure, darkness, and low temperature. The Yap and Mariana Trenches, formed by the collision of the plate, are both located in the western Pacific Ocean, and the southern end of the Mariana Trench is intersected by the north–south trending Yap Trench (Crawford et al., 1986). Yap–Mariana Junction cuts across the Mariana Ridge and Yap Ridge and is located just to the west of the Mariana Trench. The Challenger Deep is located at the southern end of the Mariana Trench as the deepest part of the trench. Related to plate subduction and compression, a series of seamounts are located on island arcs in the Yap Trench and Mariana Trench (Xu, 2021). The specific topography of seamount would affect underwater currents and create upwelling as biological hotspots in the ocean (Clark et al., 2010). Upwelling induced by seamounts could provide a higher concentration of ammonium (${\text{NH}}_{4}^{+}$) (Ma et al., 2019), thus might subsequently promote the growth of AOA and AOB, which use ammonium as a substrate for the ammonia-oxidizing process. AOA dominated by Thaumarchaeota and AOB dominated by β-Proteobacteria have been reported previously in the crust and sediment of the Takuyo-Daigo Seamount and the Ryusei Seamount (Nitahara et al., 2011, 2017). So far, most studies focused on those microbial groups were limited to a single trench, and the lack of comparison between different trenches with PNRs was still largely unknown.

In the present study, ammonia-oxidizing microbial communities in the sediments of the Yap Trench, the Mariana Trench, and their junction region were studied based on high-throughput sequencing and quantitative PCR targeting on the *amoA* gene together with the PNR measurement, in order to (1) reveal the composition, diversity, and geographical distribution pattern of the ammonia-oxidizing microbes among these regions, (2) identify the potential impacting factors, and (3) elucidate the potential contributions of AOA and AOB to the ammonia-oxidizing process. This will help to elucidate the geographical distribution and potential contributions of those ammonia-oxidizing microbes to the nitrogen biogeochemical cycling in the deep-sea biosphere.

## Materials and methods

### Sample collection and chemical analysis

**Figure 1:**
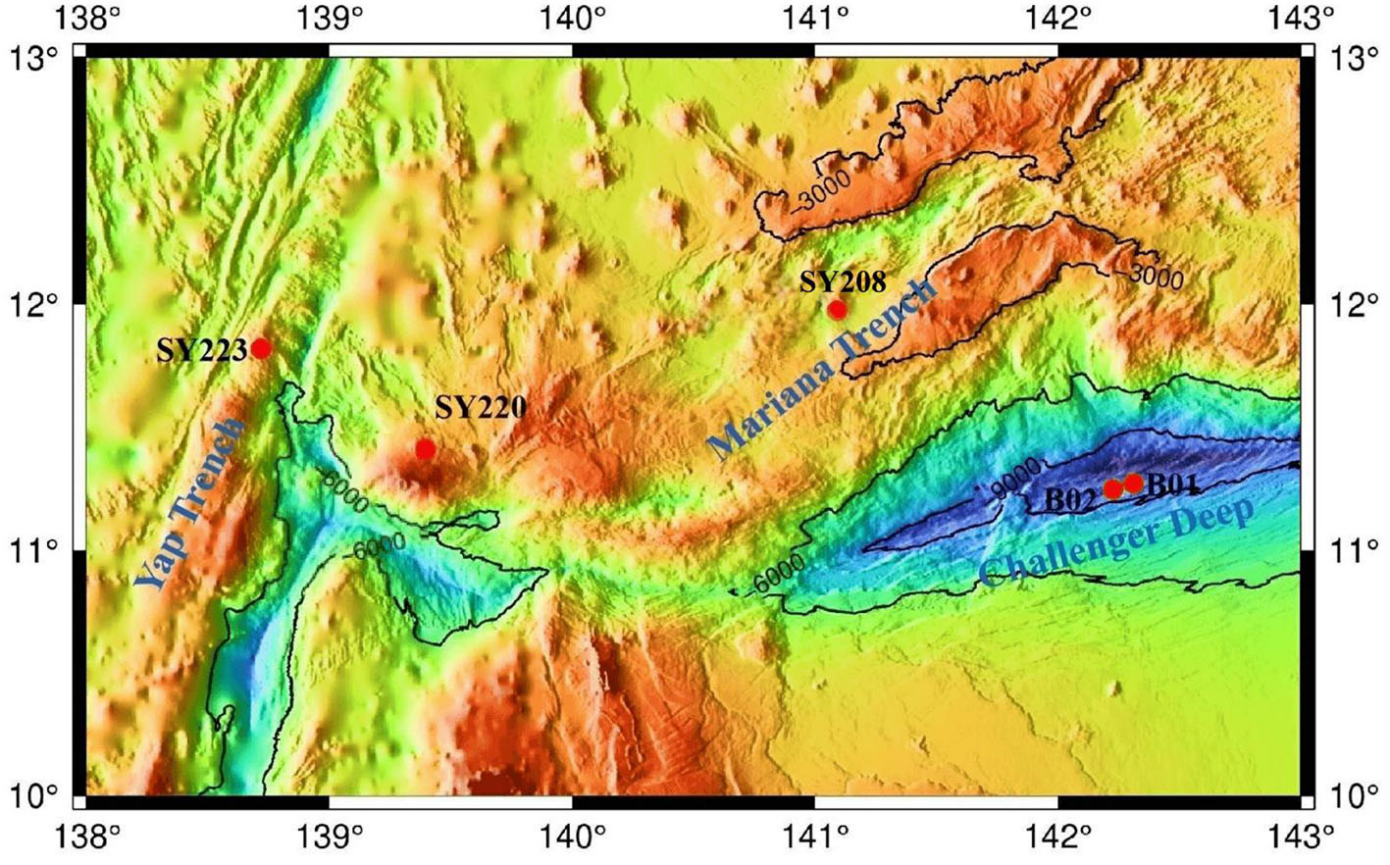
Locations of the sampling stations in the Yap and Mariana Trenches.

Sediment samples were collected using a pushcore from the Yap Trench, the Mariana Trench, and their junction region in the Western Pacific Ocean during cruise TS14 on R/V “Tan Suo Yi Hao” in 2019 (Figure 1). *In situ* hydrographical parameters (i.e., depth and location) were recorded during sampling using the manned submersible “SHENHAI YONGSHI.” The top two layers, each of 6 cm, were sliced and split into two fractions. One fraction was stored at −80°C for molecular study, and the other fraction was incubated at 4°C in the dark for potential nitrification rate measurement.

An analysis of sediment properties with ~5 g of sediment was conducted at the Institute of Mountain Hazards and Environment, Chinese Academy of Sciences (Chengdu, Sichuan, China), according to Wang et al. (2016). In brief, nitrate and ammonia were detected after 1 M HCl treatment followed by analysis with a colorimetric auto-analyzer (SEAL Analytical AutoAnalyzer 3, Germany). The concentrations of total organic carbon (TOC) and total nitrogen (TN) were determined by over-drying the sediments at 105°C and then using an element analyzer (Elementar vario MACRO cube, Germany). After digestion of the sediment, TP was measured with nitric acid–perchloric acid using the molybdate colorimetric method with a UV2450 (Shimadzu, Japan) (Murphy and Riley, 1962).

### Potential nitrification rate activity measurement

Potential nitrification rates (PNRs) for total AOA +AOB and AOA were measured with replicates in two sets of experiments (groups A and B) following the procedure by Zheng et al. (2014). For each sample, 6 g homogenized, field-moist sediment was placed in 50 ml Erlenmeyer flasks with 30 ml seawater filtered using a 0.22 μm filtration membrane. All samples were amended with 300 μM ammonium (NH_4_Cl) and 60 μM phosphate (KH_2_PO_4_). Group B was combined with a final concentration of 1.0 g·L^−1^ ampicillin to inhibit the activity of bacteria. The flasks were loosely covered and incubated at 4°C, a temperature approximating that of the bottom water, with continuous shaking in the dark. Subsamples were collected at days 0, 5, and 10 during incubation, and then, 6ml KCl (2 M) was added to extract nitrite and centrifuged and filtered for nitrite analysis. Nitrite (${\text{NO}}_{2}^{-}$) concentrations were measured using an auto-analyzer (QuAAtro, Blue Tech Co., Ltd., Tokyo, Japan). PNRs were calculated based on the changes in nitrite concentrations over time. Based on group A and group B, the total and archaeal ammonia oxidation rates were estimated through analyses of nitrite concentration changes. The bacterial ammonia oxidation rate was determined by subtracting the group B from the group A.

### cDNA synthesis, PCR amplification, and sequencing

During the PNR incubation, total RNA was extracted from ~0.7 g of the samples at the initial day (day 0, D0) and end day (day 10, D10) with the E.Z.N.A.® Soil RNA Kit (Omega Bio-Tek, Inc., Georgia, USA), according to the manufacturer's protocol. The RNA concentration was measured with a NanoDrop 2000 Spectrophotometer (Thermo Fisher Scientific Corp.). RNA was treated with DNase I (Invitrogen) and incubated at room temperature for 15 min to eliminate potential DNA contamination. The DNase I was, then, inactivated by heating at 65°C for 10 min with 1 μl EDTA. RNA (up to 200 ng) was, then, reverse-transcribed to cDNA using the SuperScript III First-Strand cDNA Synthesis Kit (Invitrogen). A parallel reaction without SuperScript III RT was used as an RT-PCR negative control. Synthesized cDNA was further digested with 1 μl RNase H at 37°C for 20 min to remove residual RNA and then used for subsequent PCR amplification. Non-reverse transcription samples were used as negative controls.

The V3–V4 region of the 16S rRNA gene was amplified via PCR with cDNA as template using primers of Bac338F (5′-TCCTACGGGAGGCAGCAGT-3′) and Bac806R (5′-GGACTACCAGGGTATCTAATCCTGTT-3′) (Oppliger et al., 2008) for bacteria and primers of Arch340F (5′-CCCTAYGGGGYGCASCAG-3′) and Arch806R (5′-GGACTACVSGGGTATCTAAT-3′) for archaea (Takai and Horikoshi, 2000). These primers were tagged with a 6 bp barcode for differentiation of amplicons in the pools of all samples multiplexed for Illumina. PCR amplification was carried out in triplicate using the BIO-RAD C1000 TouchTM Thermal Cycler PCR System in a 20 μl PCR reaction mix, containing 2.0 μl 10× PCR-MgCl_2_ buffer, 0.7 μl 2.5 mM dNTPs, 0.7 μl MgCl_2_, 0.8 μl forward primer, 0.8 μl reverse primer, 0.2 μl Platinum® TaqDNA polymerase, 2.5 μl template DNA, and 12.3 μl ddH_2_O. Thermal cycling was performed at 95°C for 3 min, followed by 33 cycles at 95°C for 0.5 min, 55°C for 45 s, 72°C for 30 s, and a final extension at 72°C for 8 min. Double-distilled water was used as a negative control. Amplification and paired-end sequencing of the amplicons were then performed with an Illumina HiSeq PE250 sequencer (Novogene Co., Ltd., www.novogene.com).

### Quantitative PCR

cDNA was used as a template for quantification of the AOA *amoA* gene during the PNR incubation with primers of amoA196F (5′-GGWGTKCCRGGRACWGCMAC-3′) and amoA277R (5′-CRATGAAGTCRTAHGGRTADCC-3′) and for quantification of the AOB *amoA* gene with primers of amoA-1F (5′-GGGGTTTCTACTGGTGGT-3′) and amoA-2R (5′-CCCCTCKGSAAAGCCTTCTTC-3′) (Leininger et al., 2006) using the StepOnePlus qPCR system (Applied Biosystems Inc., Carlsbad, CA, USA). Each qPCR reaction comprised 10 μl 2 × SYBR® Premix Ex Taq™II (Takara Bio Inc., Shiga, Japan), 0.3 μm of each primer, 1 μl DNA as the template, 0.4 μl ROX reference dye, and 20 μl water. Quantitative PCR reactions and calibrations were performed as reported before (Takai and Horikoshi, 2000). Triplicate qPCR reactions were performed for each sample with efficiencies of ~114.8% (AOA) and 110.9% (AOB), and the Ct values were calculated as gene copies against the standard curve.

### Bioinformatics analysis

After sequencing, barcodes and low-quality sequences were removed using Quantitative Insights Into Microbial Ecology 2 (QIIME 2) with default parameters (Caporaso et al., 2010). Chimeras were detected and removed with UCHIME against the SILVA database release 138 (www.arb-silva.de; Pruesse et al., 2007), and reads presented as a single copy (i.e., singletons) were removed manually. The remaining reads were, then, clustered into amplicon sequence variant (ASV) by the DADA2 (Divisive Amplicon Denoising Algorithm) algorithm. Taxonomy assignment of ASV was determined from the SILVA database release 138; ASV unrelated to prokaryotes was further removed (Caporaso et al., 2010). A filtered ASV table of each sample was generated with QIIME 2, and diversity (Shannon, Simpson, and Chao1) and Good's coverage were then calculated. For the prediction of functional and metabolic profiles of the prokaryotic community based on the 16S rRNA gene sequences, the open-source R package Tax4Fun (Asshauer et al., 2015) was used with the short-read mode disabled along with the SILVA database 138 as required. To evaluate the number of shared ASVs among the samples, a Venn diagram was generated with Venn diagram software (www.vandepeerlab.org/?q=tools/venn-diagrams). The shared ASVs were defined as core components. Distributions of archaeal and bacterial groups among the Stns. SY208, SY220, and SY223 were illustrated by ternary plots using the “ggtern” package (Hamilton and Ferry, 2018). Circos plots were generated using the “circlize” package (version 0.4.11), to show the distribution of the AOA and AOB at different stations. Network analysis was conducted to explore the co-occurrence patterns within/between the taxa of archaea and bacteria. A similarity matrix was first generated by inputting a typical ASV matrix file, and then, the correlation matrix, r-value, and p-value were calculated using corr. test in the “psych” package (Revelle, 2019) of R version 3.5.3. ASVs which are strongly and significantly correlated (Spearman′s |r| > 0.6 and FDR-adjusted *p* < 0.05) were used to construct the networks using Gephi version 0.9.3 (Bastian et al., 2009).

### Statistical analysis

The non-metric multidimensional scaling (nMDS) based on the Bray–Curtis similarity index was calculated with PRIMER 5 (Plymouth Marine Laboratory, West Hoe, Plymouth, UK) (Clarke and Warwick, 2001), to show the similarity among different samples. An analysis of similarities (ANOSIM) based on the relative abundance of ASVs was conducted with Paleontological Statistics (PAST) version 3 (Hammer et al., 2001), to test whether there was a significant difference between the microbial community structure and potential metabolic function among different samples. Values of *p* < 0.05 and *p* < 0.01 were considered to indicate different levels of statistical significance. A redundancy analysis (RDA) was performed to identify a possible differentiation of the communities under the constraint of environmental factors and assess correlations between environmental variables and community variability. The phylogenetic group data were Hellinger-transformed, environmental variables were logarithm-transformed, and the effects of collinearity (VIF > 20) were removed.

### Data available

16S rRNA gene sequences obtained from this study have been deposited in the National Center for Biotechnology Information (NCBI) Sequence Read Archive (SRA) under the accession numbers of PRJNA855595 and PRJNA855596 for archaeal sequences of D0 and D10, respectively, and PRJNA855583 and PRJNA855592 for bacterial sequences of D0 and D10, respectively.

## Results

### Geochemical characterization of the sediments

Among the five stations, Stn. SY208 was located at the Mariana with a water depth of 3,503 m, and Stn. SY220 was located in the junction between the Mariana and Yap Trenches with a water depth of 2,670 m. Stn. SY223 was located at the Yap fore arc with a water depth of 3,230 m, Stns. B01 and B02 were located at the bottom of the Challenger Deep with a water depth of ~ 10,000 m (Figure 1, Table 1), and they had significantly higher TN and TP but lower TOC and C/N ratios than other stations (*p* < 0.01, Table 1). The C/N ratios of all samples in the Challenger Deep ranging from 8.4 to 10.4 were consistent with those of the Marina Trench (Luo et al., 2017). Higher C/N ratios in the other regions might be due to the higher TOC concentrations because the TN concentrations were similar to the previous studies (Luo et al., 2017; Fu et al., 2020). No significant difference was found for the ${\text{NH}}_{4}^{+}$ and ${\text{NO}}_{3}^{-}$ contents among the three stations at the Mariana Trench (Stns. SY208, B01, and B02). Compared with the deeper layers, higher TOC and TP but lower TN and ${\text{NH}}_{4}^{+}$ contents were found in the surface layers in all the samples (Table 1).

**Table 1:** Geochemical characteristics of the sediment samples collected from the three different regions.

**Regions**	**Sample**	**Lon. ^(o^N)**	**Lat. (°E)**	**Depth (m)**	**TN (mg/kg)**	**TOC (mg/kg)**	**C/N**	**NO3- (mg/kg)**	**NH4+ (mg/kg)**	**TP (mg/kg)**
Yap Trench	SY223-S	11.82	138.72	3,230	219.91	73,963.77	336.3	1.56	1.56	542.21
	SY223-B	11.82	138.72	3,230	161.57	70,201.31	434.5	1.48	1.67	488.01
Junction	SY220-S	11.44	139.41	2,670	199.84	96,726.13	484.0	2.84	1.52	531.88
	SY220-B	11.44	139.41	2,670	196.13	90,963.77	463.8	2.01	1.63	424.21
Mariana Trench	SY208-S	11.85	141.19	3,503	218.58	102,831.94	470.5	1.41	1.87	368.52
	SY208-B	11.85	141.19	3,503	198.35	79,201.31	399.3	1.48	1.91	269.67
Challenger Deep	B01-S	11.27	142.31	9,946	441.97	3,728.71	8.4	0.92	2.05	1,087.51
	B01-B	11.27	142.31	9,946	419.54	3,349.43	8.0	1.01	2.51	912.42
	B02-S	11.25	142.23	10,063	428.51	4,438.60	10.4	0.99	2.50	1,163.07
	B02-B	11.25	142.23	10,063	408.61	4,048.33	9.9	1.08	2.63	1,076.02

Lon. and Lat. represented longitude and latitude, respectively. S and B represented the 0–6 cm and 7–12 cm sediment layers, respectively.

### Diversity and community composition of prokaryotes

High-throughput sequencing generated ~1.5 million and ~1.6 million high-quality reads for archaea and bacteria and were assigned to 222 and 1,111 ASVs, respectively, and the lowest value of ASVs was found at Stn. B01 on D0 (Table 2). For the archaeal community, Shannon diversity between the surface (0–6 cm) and the deeper (7–12 cm) layers had no significant difference at all the stations. After PNR incubation, the archaeal Shannon diversity increased in the surface layers but decreased in the deeper layers at Stns. SY223 and SY220. For the bacterial community, Shannon diversity was slightly higher on the surface than in the deep layer at all the stations but without significant difference among all stations. After PNR incubation, the bacterial Shannon diversity decreased in the surface layers but increased in the deeper layers at all the stations (Table 2, Supplementary Figure S1).

**Table 2:** Sequencing information and diversity parameters of the archaeal and bacterial 16S rRNA genes in this study.

**Microbial groups**	**Regions**	**Samples**	**Quality reads (D0)**	**ASVs (D0)**	**Shannon (D0)**	**Goods coverage (D0)**	**Quality reads (D10)**	**ASVs (D10)**	**Shannon (D10)**	**Goods coverage (D10)**
Archaea	Yap	SY223S	76,324	30	0.87	0.99	62,102	41	1.33	0.99
		SY223B	79,382	68	0.69	0.99	65,462	48	0.47	0.99
	Junction	SY220S	81,588	24	1.16	0.99	86,272	60	1.23	0.99
		SY220B	86,501	30	1.55	0.99	66,769	46	0.58	0.99
	Mariana	SY208S	78,726	68	0.91	0.99	77,041	58	1.23	0.99
		SY208B	74,147	85	0.75	0.99	78,256	40	0.58	0.99
	Challenger Deep	B01S	58,422	12	0.69	0.99	51,719	16	0.62	0.99
		B01B	51,719	38	0.62	0.99	87,410	42	0.78	0.99
		B02S	77,208	32	0.58	0.99	82,948	9	0.56	0.99
		B02B	76,918	24	0.10	0.99	80,194	17	0.89	0.99
Bacteria	Yap	SY223S	80,719	299	2.04	0.99	87,923	301	2.36	0.99
		SY223B	83,009	167	1.98	0.99	81,097	254	2.07	0.99
	Junction	SY220S	84,743	208	2.02	0.99	53,665	117	1.17	0.99
		SY220B	78,887	220	1.90	0.99	84,240	226	1.97	0.99
	Mariana	SY208S	80,487	176	1.40	0.99	80,115	183	1.31	0.99
		SY208B	88,384	153	1.11	0.99	78,084	155	0.85	0.99
	Challenger Deep	B01S	77,035	198	1.53	0.99	80,540	155	1.13	0.99
		B01B	77,723	109	1.24	0.99	81,735	109	1.38	0.99
		B02S	80,173	228	2.01	0.99	89,853	56	0.60	0.99
		B02B	83,747	56	0.72	0.99	82,409	72	0.83	0.99

S and B represent the 0–6 cm and 7–12 cm sediment layers, respectively.

**Figure 2:**
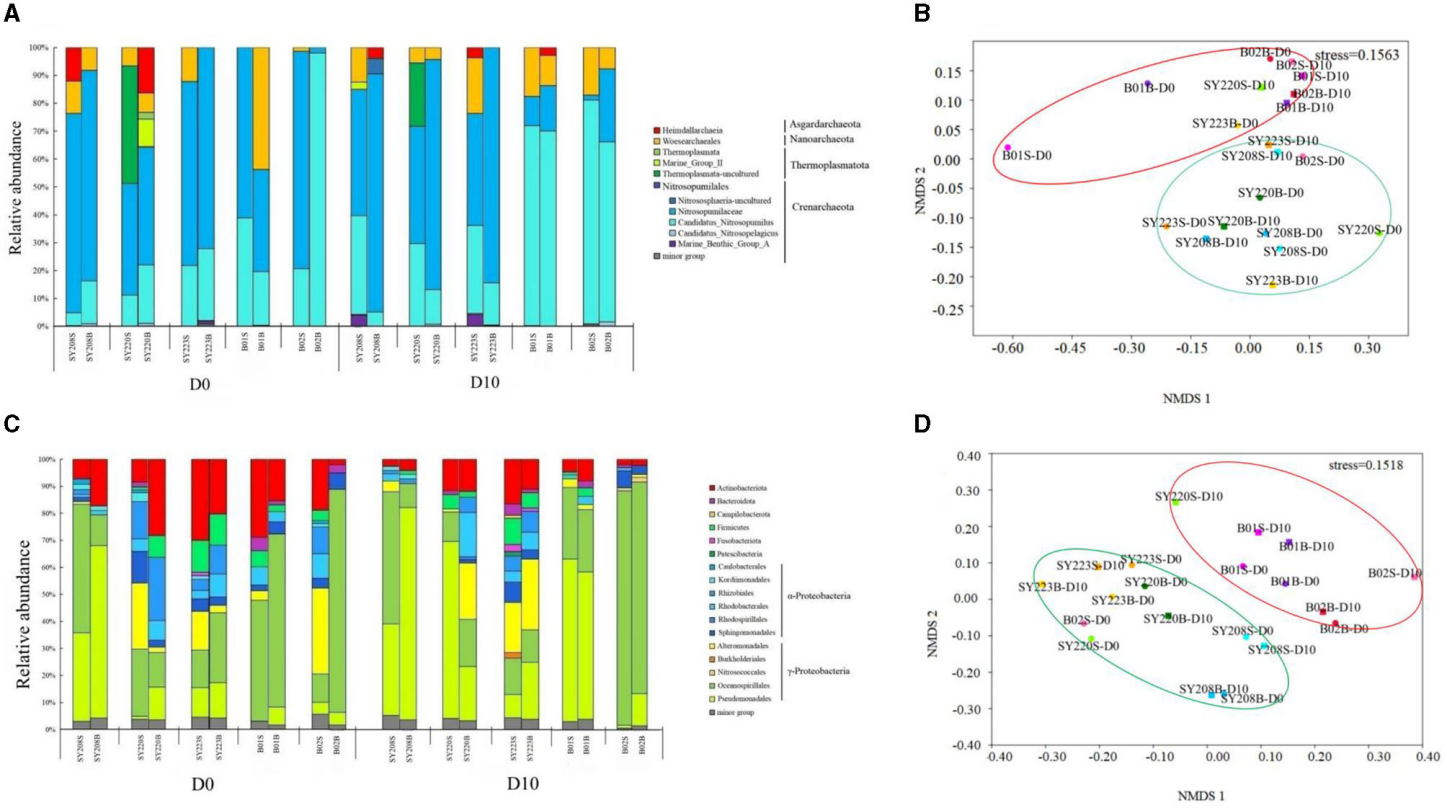
Analyses of archaeal and bacterial communities among all sediments at the initial and end of the incubation. Archaeal **(A)** and bacterial **(C)** community structures of the surface layers (0–6 cm) and deeper layers (7–12 cm) among the sediments at the phylum and class level, some at the order level with the bacterial community. Non-metric multidimensional scaling (nMDS) analysis of archaeal **(B)** and bacterial **(D)** communities among the sediments based on the Bray–Curtis distance. S and B represent the 0–6 cm and 7–12 cm sediment layers, respectively.

Crenarchaeota was the dominant archaeal phylum in all the samples, followed by Nanoarchaeota (Figure 2A). Nitrosopumilaceae and *Candidatus Nitrosopumilus* were the most abundant order of Crenarchaeota. After incubation, the proportion of Nitrosopumilaceae decreased in the surface layers but increased in the deeper layers at Stns. SY223 and SY220, while the proportion of *Candidatus Nitrosopumilus* increased in the surface layers at the two deep stations of B01 and B02. For the bacterial community, α- and γ-Proteobacteria were the dominant classes, and *Oceanospirillales* was the dominant order in all the samples (Figure 2B). After incubation, the proportion of *Actinobacteriota* significantly decreased in all samples (*p* < 0.05), while that of *Pseudomonadales* increased in all the deeper layers.

**Figure 3:**
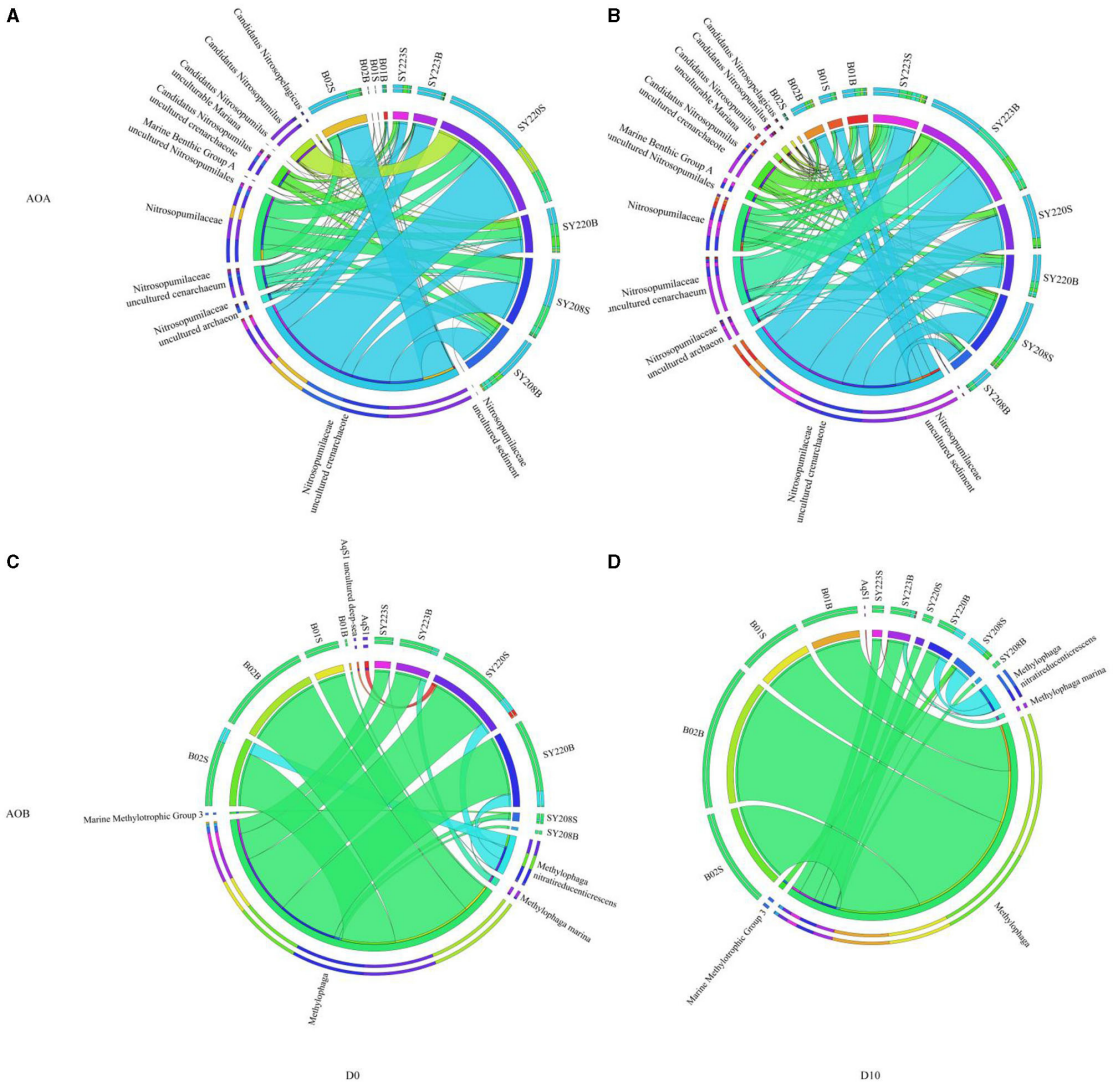
Circos plot showing the distribution of the dominant species of AOA **(A, B)** and AOB **(C, D)** among the seamount sediments of the Mariana Trench, Yap Trench, and junction regions at the initial and end of the incubation.

The distributions of AOA and AOB of different species levels were further shown with the circos plot (Figure 3). *Candidatus Nitrosopumilus* and Nitrosopumilaceae were the dominant genera of AOA in all the samples. After incubation, the proportion of Nitrosopumilaceae decreased at the Stn. SY223 and surface layers of Stns. B01 and B02 while that increased in the other stations (Figure 3A). *Methylophaga* was the dominant genus of AOB at all stations which accounted for more than 75% of each sample. Marine Methylotrophic Group 3 is only found in the surface layer of Stn. SY208. After incubation, the proportion of AqS1 increased in the deeper layer of Stn. SY223 but decreased in the surface layer of Stn. SY220 (Figure 3B).

### Spatiotemporal variation of microbial communities, environmental factors, and microbial interactions

**Figure 4:**
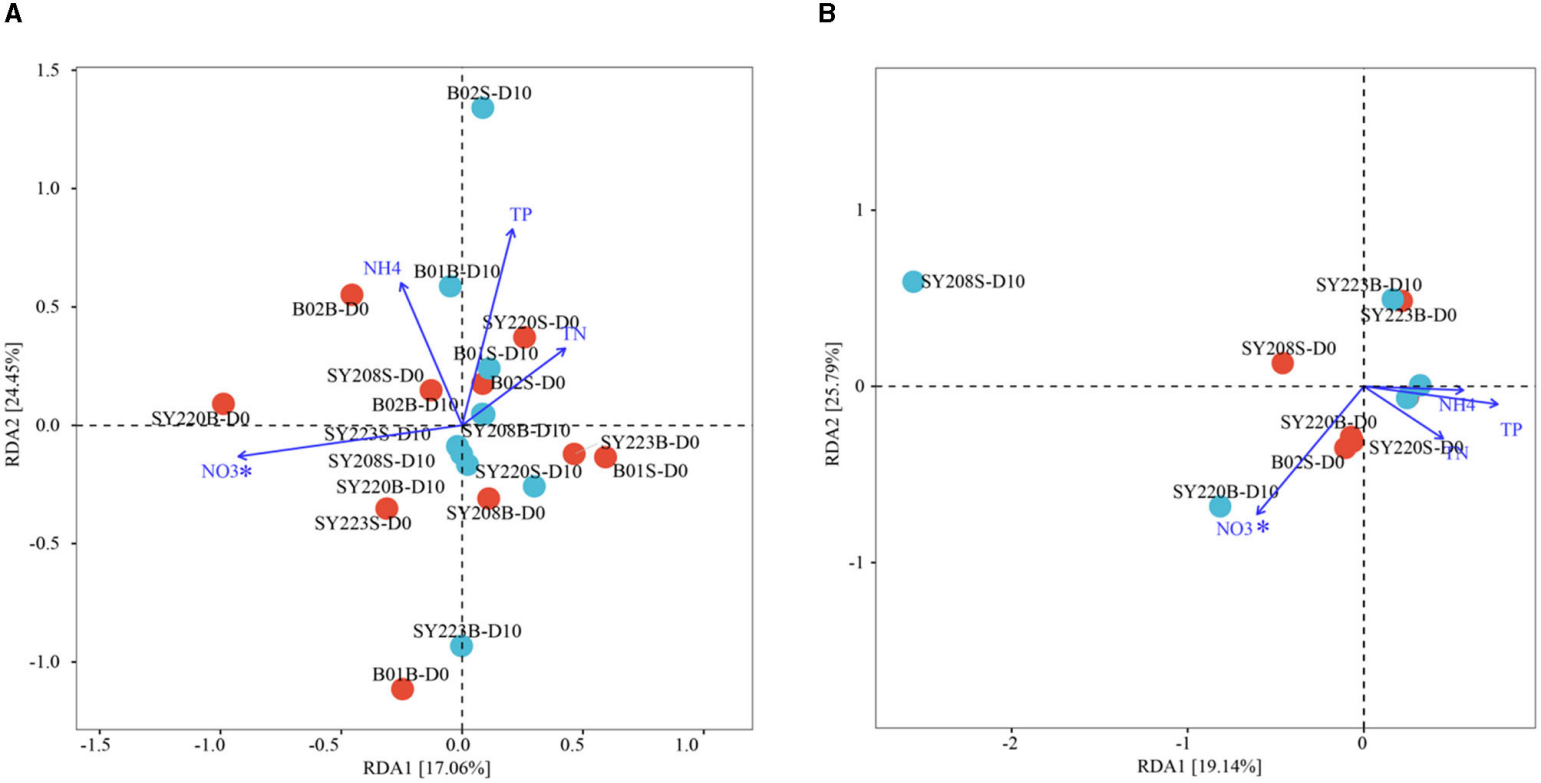
Redundancy analysis integrating the environmental parameters and the relative abundance of AOA **(A)** and AOB **(B)** at the species level among the seamount sediments of all regions at the initial and end of the incubation. (Permutation test *p < 0.05).

Based on the distribution of ASVs, the NMDS plot demonstrated that archaeal and bacterial communities at Stns. B01 and B02 were generally distinct from other stations (Figures 2C, D). The distributions of the AOA and AOB groups in the seamount sediments of Stns. SY208, SY220, and SY223 at the initial and end of the incubation were illustrated by ternary plots (Supplementary Figure S2). On the temporal scale, the AOA groups between the initial and end of the incubation were significantly different; Nitrosopumilaceae was enriched at Stns. SY208 and SY220, and *Candidatus Nitrosopumilus* was closely detected with Stn. SY220 on D0, while Nitrosopumilaceae and *Candidatus Nitrosopumilus* were significantly (*p* < 0.01) enriched at Stn. SY223 on D10 (Supplementary Figure S2). For the AOB communities, more *Methylophaga* was closely detected with Stn, SY220 on D0 while that distributed near Stns. SY208 and SY220 of the ternary plots after 10 days of incubation (Supplementary Figure S2). RDA analysis based on the AOA and AOB communities at the species level demonstrated that the ${\text{NO}}_{3}^{-}$ concentrations were the key environmental parameters that significantly influenced the AOA and AOB community structures on D0 and D10, respectively (Figure 4). The first two axes together explained 41.51% and 44.93% of the total variance of the AOA and AOB communities, respectively (Figure 4).

The network diagram analysis of the interaction between archaea and bacteria showed that the addition of substrate could significantly enhance the interaction between microorganisms and initial samples (Supplementary Figure S3). Most microorganisms had a positive correlation with D0, and only *Nitrosopelagicus* and *Kordiimonadales* had a negative correlation. After 10 days of incubation, the negative correlation was mainly observed between archaea and bacteria (Supplementary Figure S3).

### Potential nitrification rates and *amoA* transcript abundance

**Figure 5:**
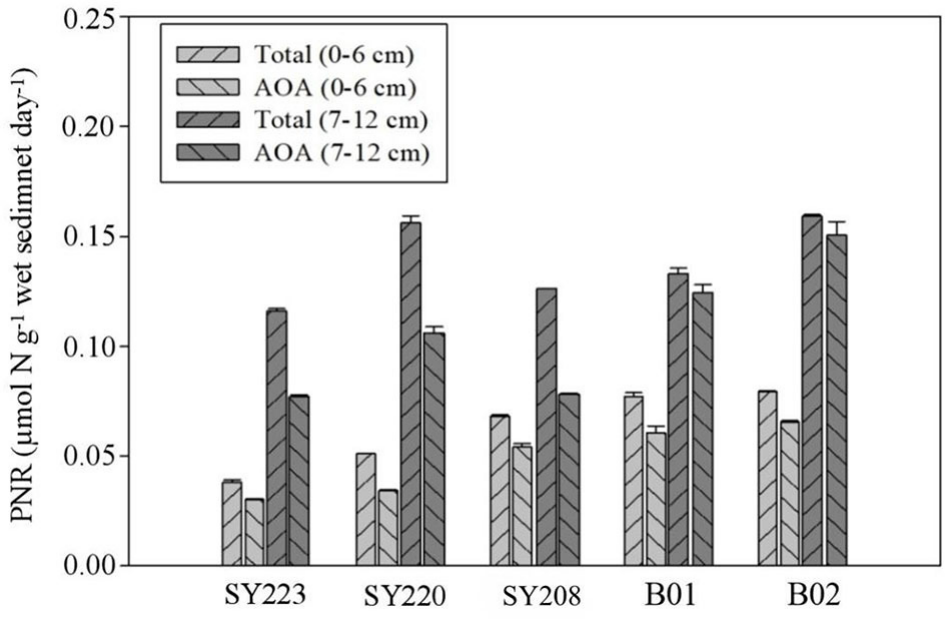
Potential nitrification rates of total AOA+ AOB and AOA in the surface (0–6 cm) and deeper layers (7–12 cm) among different sediments of all regions.

In all the samples, AOA PNRs accounted for more than 60% of the total PNRs, and both the total and AOA PNRs were significantly higher in the deeper layers than in the surface layers (*p* < 0.01). In the surface layers, they were significantly higher at the Stns. SY208, B01, and B02 in the Mariana Trench than the other stations (*p* < 0.01). As for AOB PNRs, no significant difference was found between the surface and deeper layers at all the stations. The AOB PNRs in the deeper layers of the Yap Trench (Stn. SY223) and junction regions (Stn. SY220) were significantly higher than those in the Mariana Trench (Stns. SY208, B01, and B02, *p* < 0.05, Figure 5).

**Figure 6:**
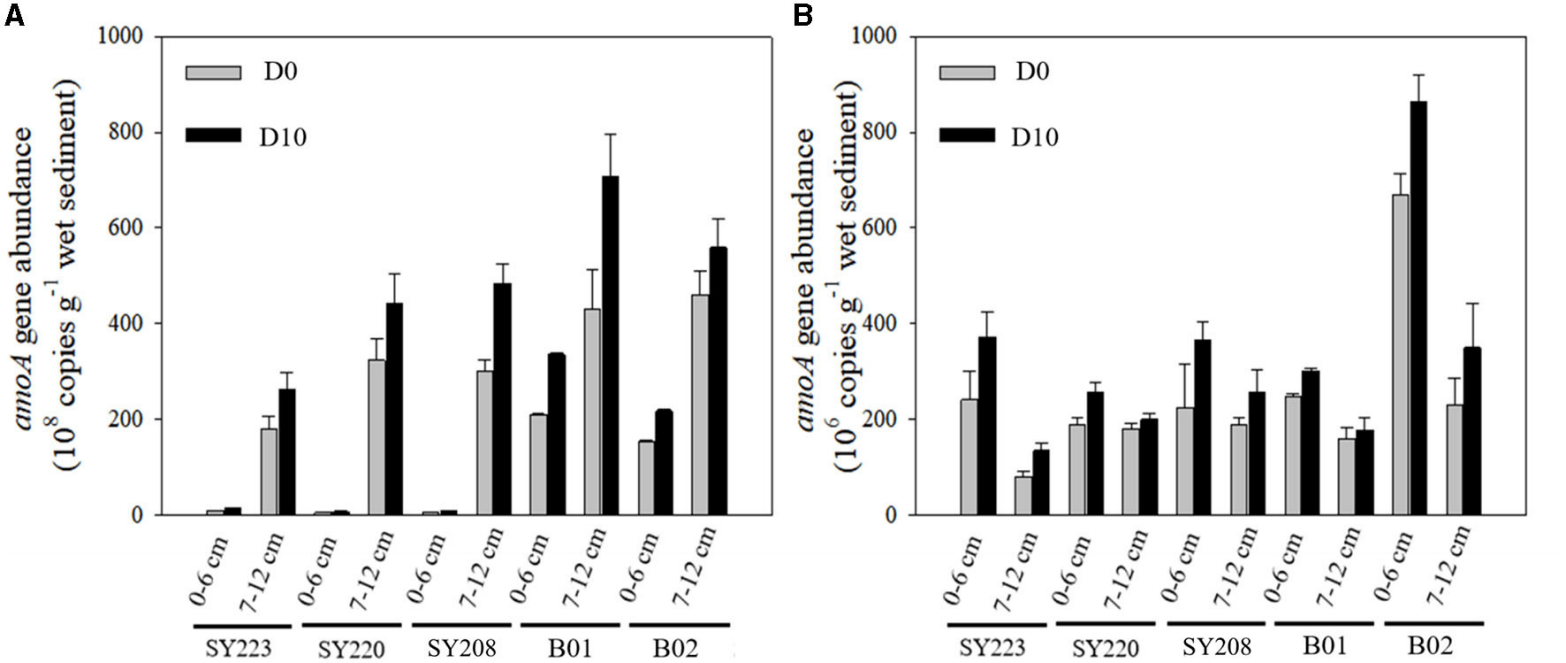
Abundances of AOA **(A)** and AOB **(B)** *amoA* gene in the surface (0–6 cm) and deeper layers (7–12 cm) in the sediments of all regions at the initial and end of the incubation.

Based on qPCR analysis of the abundance of the *amoA* gene at the cDNA level, the AOA transcripts were approximately two magnitudes higher than those of AOB in all the samples, except the surface layer of the Yap Trench (Stn. SY223) and junction regions (Stn. SY220) (Figure 6). AOA transcripts were significantly higher in the Challenger Deep (Stns. B01 and B01) than in the seamounts (Stns. SY223, SY220, and SY208, *p* < 0.05). AOA transcripts increased with the incubation time (Figure 6A) and were significantly higher in the deeper layers than in the surface layers at all the stations (*p* < 0.01), especially at the Stns. B01 and B02. For AOB transcripts, they were significantly higher in the surface layers than in the deeper layers at all stations (*p* < 0.01), and no significant difference was found among different stations. It also increased with incubation time, and more increase was observed in the surface layer than the deeper layer in the seamount sediments of the Yap Trench and junction regions (Figure 6B). Correlation analysis showed that TN and TOC had significant positive and negative effects on the distributions of the AOA *amoA* gene transcripts in the surface layers, respectively (*R* = 0.98, *p* < 0.01; *R* = −0.96, *p* < 0.05), while ${\text{NH}}_{4}^{+}$ concentration was positively correlated with the AOB *amoA* gene transcripts in the surface layers (*R* = 0.88, *p* < 0.05; Supplementary Table S1).

## Discussion

### Microbial community composition and microbial interaction

The predominance of bacteria, especially α- and γ-Proteobacteria, at all the stations was consistent with the finding in Southern Yap Trench (Fu et al., 2020), where they were involved in detrital carbon recycling (Cui et al., 2019). Rhodobacterales belonging to α*-*Proteobacteria were the main degraders of transparent exopolymer particles (Taylor and Cunliffe, 2017) and chitins (Enke et al., 2018). Alteromonadales belonging to γ-Proteobacteria could secrete a series of extracellular enzymes to degrade and utilize various organic substances and play a key role in the mineralization and biogeochemical transformation of sinking particulate organic matter in the deep ocean (Boeuf et al., 2019). These groups accounting for a higher proportion of the sediments might be related to their important roles in the carbon cycling in the marine ecosystem. This might be the reason that total organic carbon content was identified as the key environmental factor affecting the bacterial community composition in this study and further proved the importance of this factor in regulating the distribution and abundance of the bacterial community in the marine systems (Hu et al., 2021). The dominance of Nitrosopumilaceae and *Candidatus Nitrosopumilus* in the archaeal commun ity reflected the importance of the ammonia oxidation process potentially occurring in the trench sediments, possibly induced by the ${\text{NH}}_{4}^{+}$ contents. *Woesearchaeales* were widely distributed in all regions in our study, and they have been reported as the second most abundant archaeal group in the sediments of the Challenger Deep (Cui et al., 2019) and existed in the sediments of the Yap Trench (6,578 m, Zhang et al., 2018) and Izu–Ogasawara Trench (9776 m, Hiraoka et al., 2020) as well. They had the potential function of organic matter fermentation to produce acetic acid, hydrogen, and methyl compounds, subsequently supporting methane production (Liu et al., 2018). *Methylophaga* has been found from a variety of environments, including coastal marine water (Janvier et al., 1985; Neufeld et al., 2007), the sediment of the Mariana Trench (Wei et al., 2022), and *Paardenmarkt* site in the Belgian part of the North Sea (Kundu et al., 2023), with ammonium, nitrate, and urea as the typical nitrogen sources (Janvier et al., 1985). After 10-day incubation, the AOA Shannon diversity was higher at the Challenger Deep, which might be caused by the lower C/N ratios of the sediment as suggested in the previous study (Wang et al., 2020).

Microbial interactions could lead to a series of competitive or collaborative relationships and have been proposed as biotic drivers that impact microbial community composition (Hunt and Ward, 2020). Most microorganisms had a positive correlation at D0, and only *Candidatus Nitrosopumilus* and *Kordiimonadales* had a negative correlation; the former could use ammonia oxidation as an energy source for growth (Vuillemin et al., 2019), the latter had no genes involved in ammonia oxidation and nitrite reduction, but it played an important role in organic nitrogen during ammonia assimilation (Reitzer and Magasanik, 1986) and had survival advantages in ammonium-deficient environments (Geng et al., 2022). The negative correlation of them might be caused possibly induced by the ${\text{NH}}_{4}^{+}$ contents on D0. Microbial interaction was more complex on D10, and the negative correlation was mainly observed between archaea and bacteria, such as Crenarchaeota and *Pseudomonales*, Crenarchaeota and Alteromonadales, and Asgardarchaeota and Actinobacteriota. The negative correlation of microbial groups has been frequently observed as an ecological strategy in extreme environments such as Arctic deep-sea, hydrothermal vent, and Mariana Trench (Bienhold et al., 2012; Flores et al., 2012; Wang et al., 2022). The absence of Asgardarchaeota from the Yap Trench was in agreement with the previous study (Fu et al., 2020) but still deserves further investigation. Those microbial groups were very likely worked with competition, contributing to the degradation of marine organic matter in the sediments with a negative correlation. Venn diagrams showed overlapping of ASVs among different samples, and microbial groups belonging to the shared ASVs were defined as core components (Supplementary Figure S4). Six different KEGG metabolism categories, i.e., carbon fixation, carbohydrate, amino acid, nitrogen, sulfur, and lipid metabolism, were assigned for the extracted core microbial groups (Supplementary Figure S5). Generally, nitrogen metabolism was the most abundant functional category for both archaeal and bacterial groups and accounted for a relatively higher proportion at Stns. SY223 and SY220. For archaeal groups after incubation, the proportion of the nitrogen metabolism increased in the surface layers but decreased in the deeper layers at Stns. SY208, B01, and B02 in the Mariana Trench (Supplementary Figure S5).

### Biogeographical distribution and impacting factors

Distinct archaeal and bacterial communities in the Challenger Deep from other stations were observed in this study. This clear biogeographic distribution patterns might because that the deepest point of trench acted as unique niche directly or indirectly enrich the ammonium resulted from sediment fermentation (Oguri et al., 2022). On the other hand, samples of Stns. SY208, SY220, and SY223 were from the base of the seamounts, where the enriched concentrations of particle organic matter subsequently support the development of distinct microbial structure from the surrounding oligotrophic environments (Mendonca et al., 2012). In addition, in our study, higher diversity and more endemic species were formed in the seamounts than in the Challenger Deep. Significantly higher bacterial abundance and biomass were detected in the sediments of the Marsili and Palinuro seamounts than in the adjacent plain sediments (Danovaro et al., 2009). This might be attributed to enclosed circulation cells created by the seamount (White et al., 2007), which reduced community connectivity between the seamount and the surrounding areas (Giljan et al., 2020). Such seamount effect would cause microbial diversification, resulting in greater microbial diversity and species richness around seamounts than in the surrounding areas. Sediments might restrict the dispersal of species; thus, the seamount effects might not be so obvious for the pelagic communities. Detailed information about the topographic and hydrodynamic features of seamounts would be helpful in disentangling the seamount effects on shaping the microbial communities in this extreme biosphere in future studies. Along the vertical niches, higher bacterial community diversity was shown in the surface layers than in the deeper layers, which might be due to nutrient content, especially TOC and TN, which decreased with an increased depth. A decrease in microbial community diversity with different sediment layers in the Yap Trench was also reported previously (Fu et al., 2020). This result might come from the decreased TOC and TN contents. With fewer nutrients in the sediments and longer burial time in the deeper layers, the abundance of total microbes decreased and microbial degradation increased in hadal sediments.

Interestingly, in our study, the most important factors that significantly influenced the spatial and temporal variation of the AOA and AOB communities were the ${\text{NO}}_{3}^{-}$ concentration but not the ${\text{NH}}_{4}^{+}$ concentration. This might be due to the fact that not all AOA were actively involved in ammonia oxidation (Church et al., 2010); some Nitrospira strains performed complete oxidation of ammonia to nitrate by the comammox process, and nitrite oxidizers could be involved in complex associations with different microorganisms (Cabello et al., 2019). Furthermore, a previous study showed that nitrate concentration in the water column was tightly controlled by the abundance of ammonia-oxidizing archaea and bacteria (Shan et al., 2023). Meanwhile, a low C/N ratio in the cathode layer beneficial to the dominant AOA (Wang et al., 2020) might have the same effects as the samples on the Challenger Deep.

### PNRs and *amoA* gene abundance among different regions

Potential nitrification rates can reflect potential ammonia-oxidizing activity (He et al., 2018). The PNRs detected in the current study were remarkably lower than those in the sediments of estuaries (Li et al., 2015; Santos et al., 2018). This is very likely due to the lower ${\text{NH}}_{4}^{+}$ concentration and temperature in deep-sea sediments because these two factors were known to have significant effects on the PNRs (He et al., 2018). The finding that AOA contributed more to the PNRs in those deep-sea sediments was consistent with the report in low-ammonium open ocean (Mincer et al., 2007) and was very likely due to the high sensitivity of AOA to ammonia than AOB (Prosser and Nicol, 2012). We found the PNRs in Challenger Deep and seamounts at the same level, and this could be due to the process in different environments being driven by different environmental factors to finally reach a similar level. Higher ${\text{NH}}_{4}^{+}$ concentration found at the Mariana Trench could provide substrates for the PNRs, while in the seamount sediment regions, significantly higher TOC content decomposed by microorganisms could supply re-mineralized ${\text{NH}}_{4}^{+}$ as a continuous source for PNRs (Fu et al., 2020). The promotion of AOA and AOB transcripts with the addition of ${\text{NH}}_{4}^{+}$ has been reported (Li and Gu, 2013). However, increasing evidence suggested that marine AOA (i.e., *N. maritimus* strains) could utilize organic nitrogen (i.e., urea and cyanate) as the substrates of nitrification (Kitzinger et al., 2019). Additionally, significantly higher AOA PNRs in the deeper layers than in the surface layers at all the stations might be due to less dissolved oxygen and higher ${\text{NH}}_{4}^{+}$ concentration existing in the deeper layers.

Regarding the abundance of the *amoA* gene transcript, in this study, AOA overrides AOB which was in agreement with previous studies in the Eastern Tropical North Pacific Ocean and Yap Trench (Beman et al., 2012; Fu et al., 2020). Significantly higher abundance of AOA transcripts existed in the Challenger Deep (Stns. B01 and B02) than in the seamounts, and this might be due to the low *amoA* gene abundance corresponding to high total organic carbon (TOC) (Yang et al., 2016), although seamounts were prosed to provide many unique niches for microbes and promote their metabolic abilities as well (Rogers et al., 2010). There was no significant difference in *amoA* gene transcript among the different seamounts, and this might be due to the similar nutritional levels, especially TOC, TN, and ${\text{NH}}_{4}^{+}$ in these seamount sediments. Vertically, higher AOA *amoA* gene transcripts and PNRs in the deeper layer might be because they had better adaptation to the oligotrophic conditions with low oxygen and low nutrients (Takano et al., 2010). No significant difference in AOB gene transcripts was observed among all regions, and this was in agreement with the homogenous distribution of AOB gene abundance in surface sediments of the Mariana and Yap Trenches (Nunoura et al., 2018; Fu et al., 2020). A higher abundance of AOA transcripts in the deeper layers could be attributed to its capability to outcompete AOB in the oxygen-limited deep-sea sediments (Francis et al., 2005; Jing et al., 2022), therefore leading to significantly higher PNRs in the deeper layers, especially in the Challenger Deep. AOA and AOB transcripts at all the stations increased with the incubation time, and this was possibly induced by the extra substrates that were added during the incubation experiments.

Ammonia oxidation is the first and rate-limiting step of nitrification and is critical to the nitrogen cycling in the oceans. Compared with those shallow oceanic environments, research about the relative importance of those nitrifiers in the deep trenches and seamounts is still rare. Our study proved that AOA played a predominant role than AOB in the ammonia-oxidizing process that occurred in those regions, providing a snapshot of ammonia-oxidizing microbes and associated ecological processes in those deep sediments. Future studies using a combination of metagenomics/transcriptomics with the isotopic tracing methods would be necessary to provide insights into the ecological contribution of different ammonia-oxidizing microbes to the nitrification process and nitrogen flux of the deep biosphere.

## Data availability statement

The datasets presented in this study can be found in online repositories. The names of the repository/repositories and accession number(s) can be found in the article/Supplementary material.

## Author contributions

HL: Methodology, Visualization, Writing—original draft. HJ: Conceptualization, Data curation, Funding acquisition, Investigation, Resources, Writing—review and editing. FW: Methodology, Visualization, Writing—original draft.
