# Supplementary material for: Archaea predominate in the ammonia oxidation process in the sediments of the Yap and Mariana Trenches
Source: Front Microbiol. 2023 Sep 28;14:1268790. doi: 10.3389/fmicb.2023.1268790 (PMC10568479; doi:10.3389/fmicb.2023.1268790)
Supplement: Supplementary file 1 [file Data_Sheet_1.pdf]

Supplementary Materials

Table S1. Pearson correlations between the abundances of AOA and AOB *amoA* gene in different sediment layers (7-12 cm) and environmental factors. NS represented no significant correlation. S and B represented 0-6 cm and 7-12 cm layers, respectively.

amoA gene abundance	TN	TOC	C/N	NO_3^-	NH_4^+	TP
AOA -S	R = 0.9786, p = 0.0037	R = -0.9581, p = 0.0102	R = -0.9439, p = 0.0158	NS	NS	R = 0.9433, p = 0.0161
AOA-B	NS	NS	NS	NS	NS	NS
AOB-S	NS	NS	NS	NS	R = 0.8802, p = 0.0489	NS
AOB-B	NS	NS	NS	NS	NS	NS

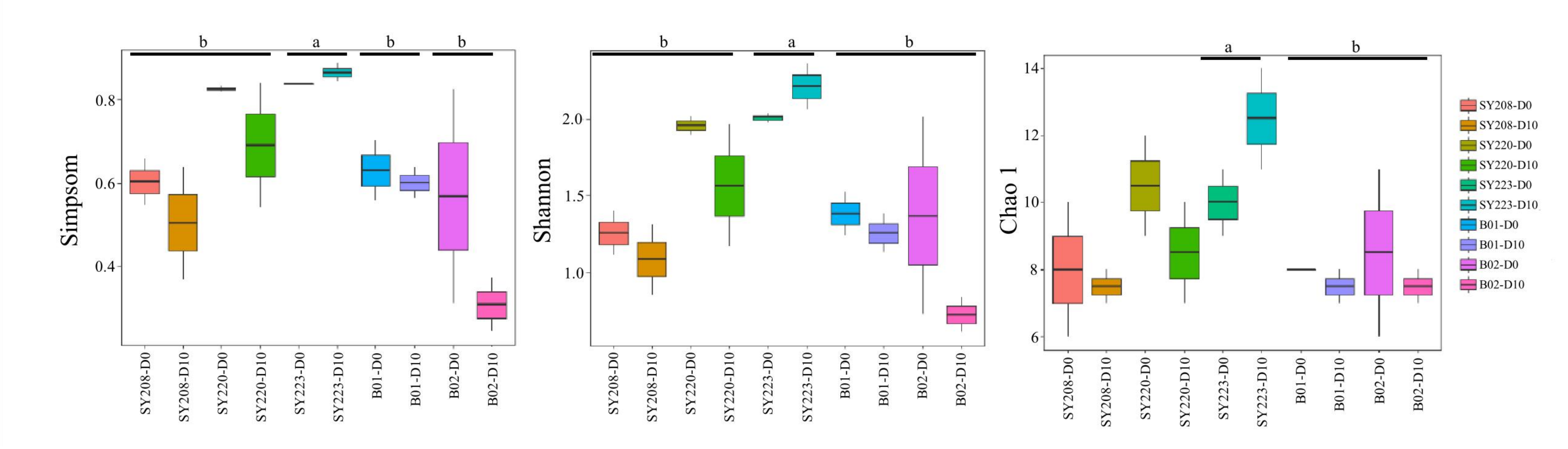

Figure S1. Alpha diversity indices (Simpson, Shannon and Chao1) of bacterial communities of the surface (0-6 cm) and deeper layers (7-12 cm) in sediments of all samples at the initial and end of the incubation. ab, $p < 0.05$.

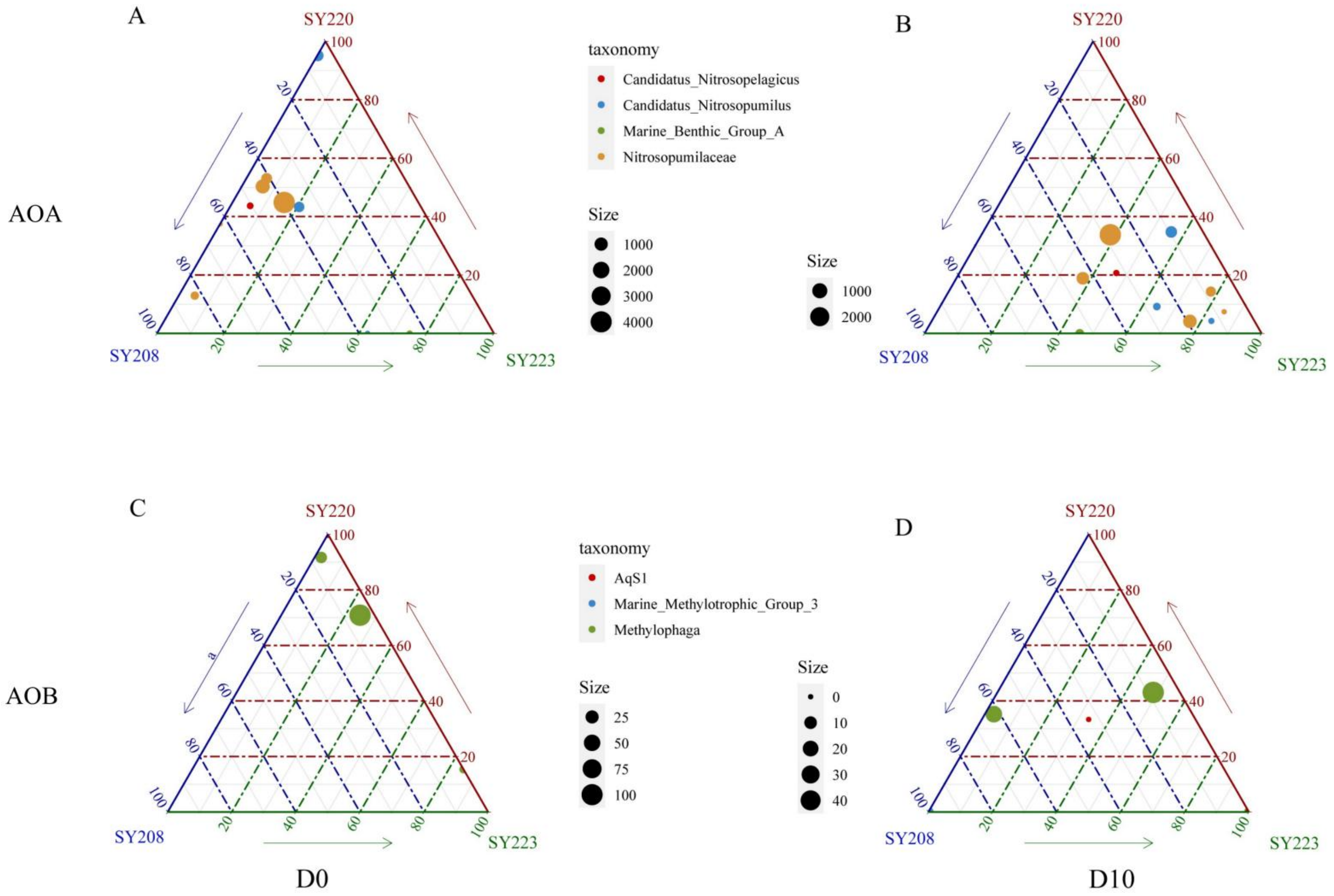

Figure S2. Ternary plots of AOA (A, B) and AOB (C, D) assemblages among the seamount sediments of the Mariana, Yap Trench and junction regions at the initial and end of the incubation.

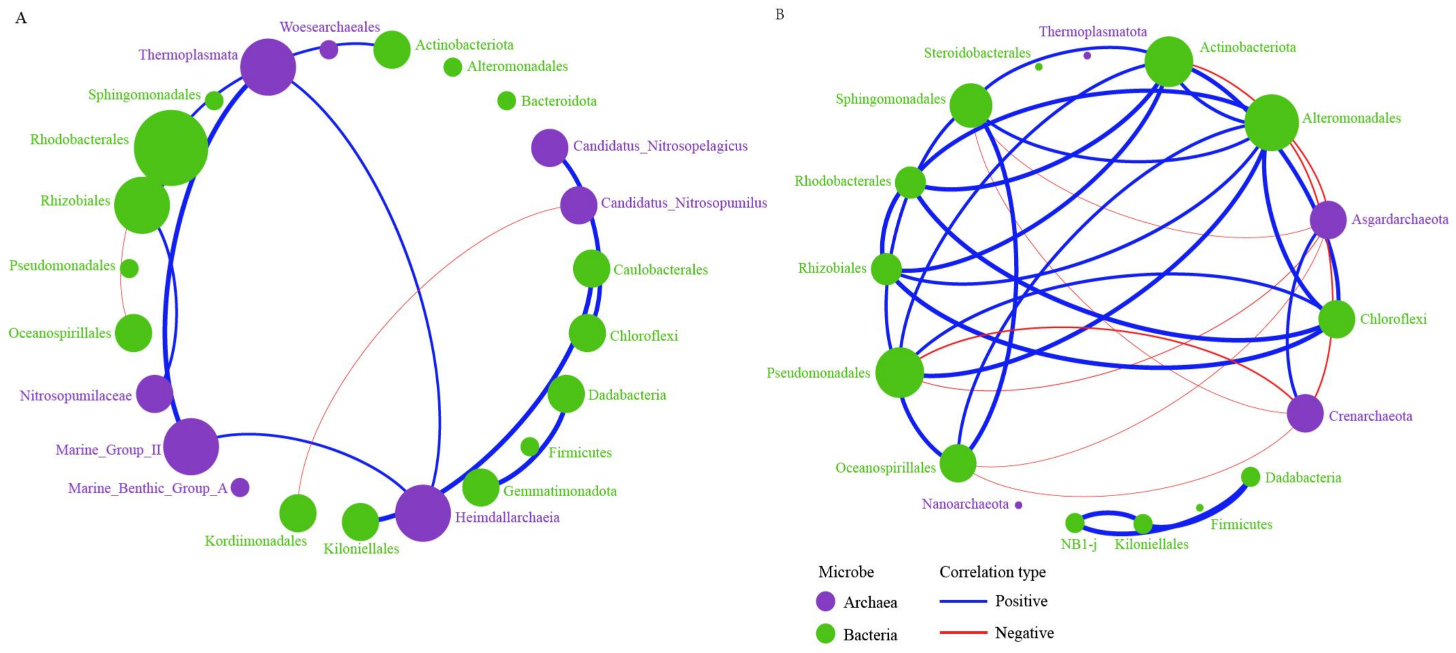

Figure S3. The networks analysis of archaea and bacteria in all sediments at D0 (A) and D10 (B) of the incubation. The networks represent relationships between co-occurring ecosystems and the edges represent co-occurrence relationships consistent at the 0.6 correlation level. The nodes represent archaeal and bacterial taxa.

.

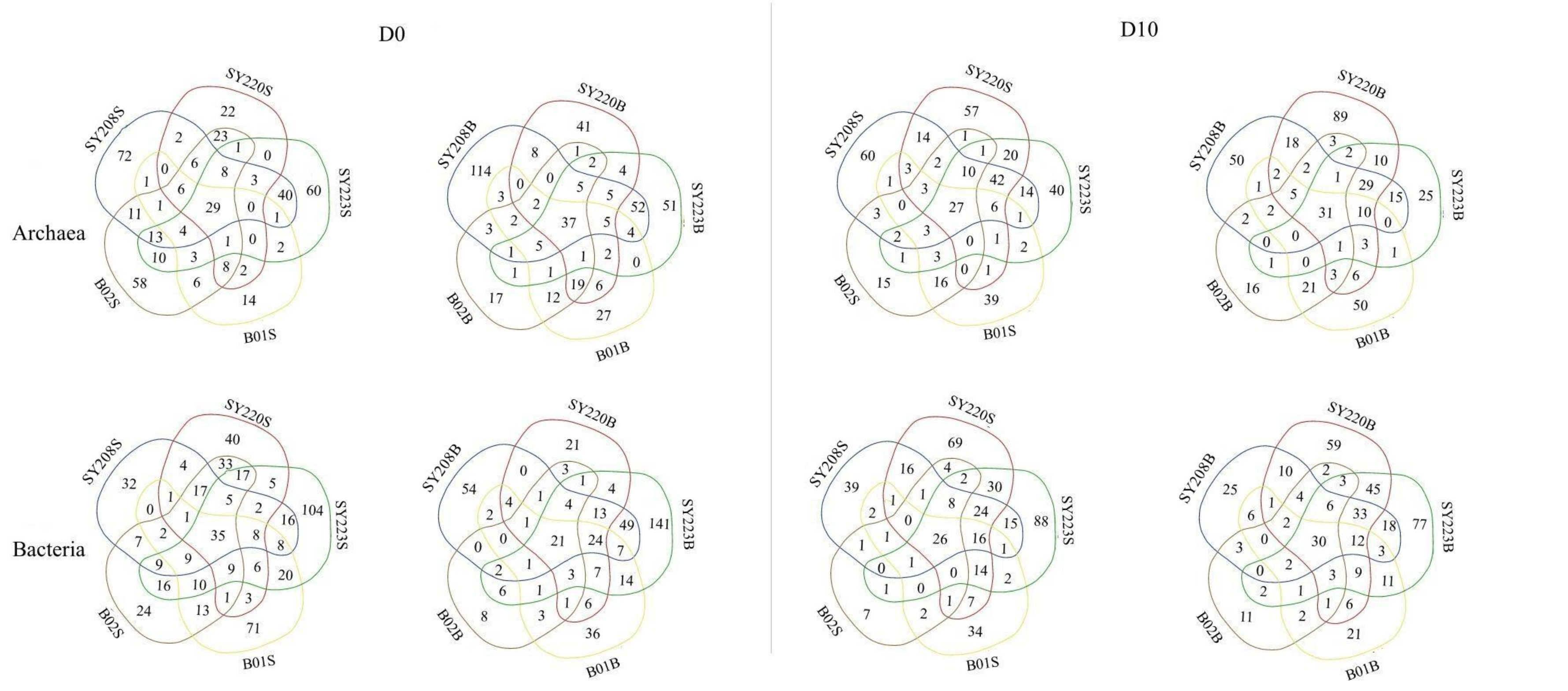

Figure S4. Venn diagrams representing the overlap of ASVs among archaeal and bacterial communities among all sediments at the initial and end of the incubation.

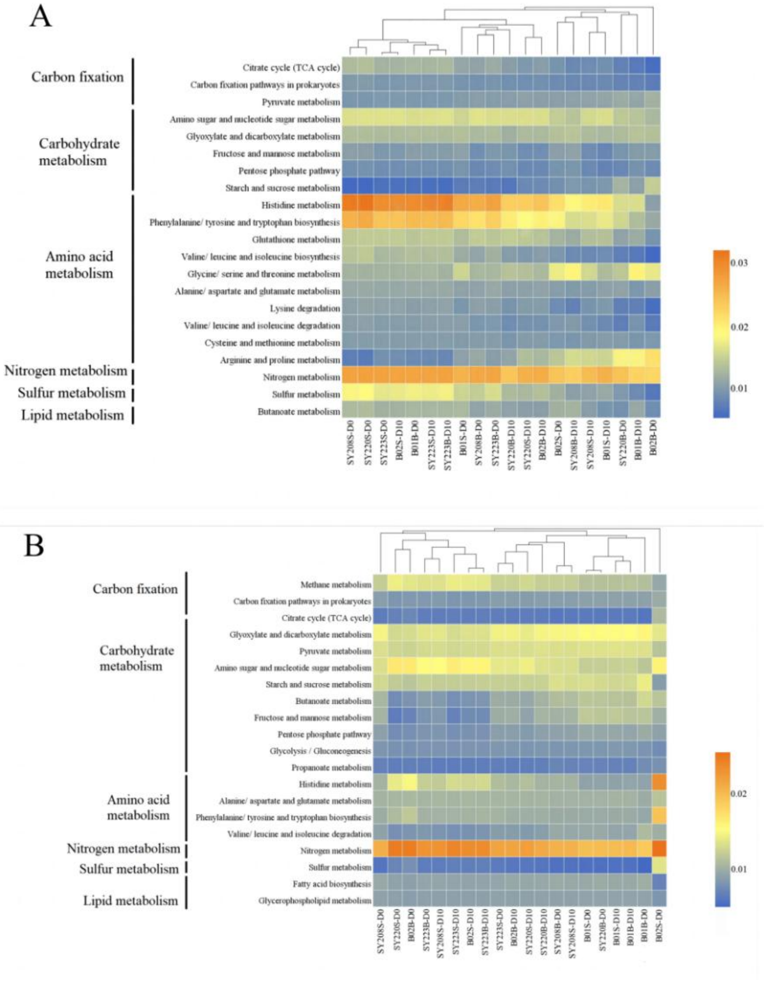

Figure S5. Heatmap and clustering of the potential metabolic categories of the core archaeal (A) and bacterial (B) ASVs at the initial and end of the incubation based on the relative abundance of functional gene referred from KEGG metabolism modules.
